# RiceReceptor: The Cell-Surface and Intracellular Immune Receptors of the *Oryza* Genus

**DOI:** 10.3390/genes16050597

**Published:** 2025-05-18

**Authors:** Baihui Jin, Jian Dong, Xiaolong Hu, Na Li, Xiaohua Li, Dawei Long, Xiaoni Wu

**Affiliations:** 1 Faculty of Agronomy and Life Science, Kunming University, Kunming 650201, China; jbhxkyz521@kmu.edu.cn (B.J.); shin777@163.com (X.H.); 2 College of Plant Protection, Yunnan Agricultural University, Kunming 650201, China; dongjian9722@163.com; 3 Pu’er Agricultural Science Research Institute, Pu’er 665000, China; lina6550@126.com (N.L.); lxh9617_1@126.com (X.L.); 4 Healthcare School, Tacheng Vocational and Technical College, Wusu 834700, China; duoerguen313@126.com

**Keywords:** RiceReceptor, leucine-rich repeat, nucleotide-binding leucine-rich repeat, biotic stress, resistance

## Abstract

Introduction: Rice, a cornerstone of global food security, faces escalating demands for enhanced yield and disease resistance. We collected 300 high-quality genomes, representing both cultivated (*Oryza sativa indica*, *O. sativa japonica*, and *O. sativa aus*) and wild species (*O. rufipogon*, *O. glaberrima*, and *O. barthii*). Methods: Leveraging HMMER, NLR-Annotator, and OrthoFinder, we systematically identified 148,077 leucine-rich repeat (LRR) and 143,459 nucleotide-binding leucine-rich repeat (NLR) genes, with LRR receptor-like kinases (LRR-RLKs) dominating immune receptor proportions, followed by coiled-coil domain containing (CNL)-type NLRs and LRR receptor-like proteins (LRR-RLPs). Results: Benchmarking Universal Single-Copy Orthologs (BUSCO) assessments confirmed robust genome quality (average score: 94.78). Strikingly, 454 TIR-NB-LRR (TNL) genes—typically rare in monocots—were detected, challenging prior assumptions. Phylogenetic analysis with Arabidopsis TNLs highlighted five *O. glaberrima* genes clustering with dicot TNLs; these genes featured truncated PLN03210 motifs fused to nucleotide-binding adaptor shared by APAF-1, R proteins, and CED-4 (NB-ARC) and LRR domains. Conclusions: By bridging structural genomics, evolutionary dynamics, and domestication-driven adaptation, this work provides a foundation for targeted breeding strategies and advances functional studies of plant immunity in rice.

## 1. Introduction

Rice is one of the most important staple crops globally, providing essential food for hundreds of millions of people [1,2,3]. With the continuous growth of the world population, there are increasing demands for both the yield and quality of rice and other cereal crops, making food security a significant challenge faced by the global community [4,5]. In this context, rice production encounters various complex stress factors, which can be broadly categorized into biotic and abiotic stresses. Biotic stresses primarily arise from pathogenic microorganisms, such as bacteria, fungi, and viruses, as well as pest infestations [6,7]. These biotic factors not only affect the growth and development of rice but can also lead to significant yield losses and declines in quality [8]. Research indicates that the losses caused by biotic stresses are substantial and may severely impact farmers’ livelihoods and national food security [9]. Therefore, understanding the mechanisms by which rice responds to these biotic stresses is crucial for enhancing its resistance and productivity.

Under biotic stress, both cell-surface immune receptors and intracellular immune receptors play important roles [10,11,12]. Cell-surface immune receptors are capable of recognizing features of external pathogens and rapidly initiating defense mechanisms to prevent pathogen invasion [11]. In contrast, intracellular immune receptors are responsible for monitoring internal cellular changes; upon detecting pathogen invasion or cellular damage, they activate a series of signaling pathways that mobilize the cell’s defense system [13]. The interactions and coordinated responses of these immune receptors constitute a complex defense network in rice against biotic stresses, ensuring its survival and reproduction in adverse environments [14,15].

Due to the rapid development of second-generation technology, an increasing number of rice planting resources have been sequenced at the genomic level [16,17]. This groundbreaking development establishes a comprehensive genomic repository for the scientific community, thereby catalyzing transformative progress in molecular breeding programs and targeted trait enhancement initiatives for *Oryza sativa* [18]. With the rise and continuous development of third-generation sequencing technology, the precise sequencing and assembly of rice genomes are becoming more prevalent [19]. The application of these new technologies enables researchers to obtain longer reads and higher sequencing accuracy, effectively overcoming the limitations of traditional sequencing methods in complex genomes [20]. Through these advanced sequencing technologies, more and more rice genomes are being accurately sequenced and assembled, allowing researchers to gain deeper insights into the genetic diversity and adaptive traits of rice [1,2]. This provides an important basis for the large-scale and accurate identification of immune receptor-related genes in rice [21]. Immune receptors play a crucial role in rice’s defense against pathogen invasion and its response to environmental stresses [22]. Therefore, the identification and functional analysis of these genes will significantly advance the progress of disease-resistant breeding in rice. Furthermore, the identification of these genes will offer new perspectives for revealing the immune mechanisms in rice and for comparative studies with other crops [23].

We collected 300 high-quality genome assemblies spanning the *Oryza* genus, comprising both cultivated rice (*O. sativa*) and diverse wild progenitor species. From these 300 genomes, we identified a total of 148,077 LRR genes and 143,459 NLR genes. The numbers of these two types of genes are similar across different rice subgroups. The LRR and NLR genes in rice display considerable variation in terms of their number and function. These genes serve as essential elements of the rice immune system by engaging in intricate interactions, which offer both broad-spectrum and specific resistance to diseases in rice [24,25]. Additionally, TNL genes are crucial in the plant’s disease resistance process, as they detect pathogen effectors and trigger appropriate responses [24]. TNL proteins function as signaling hubs by recruiting various proteins to form large protein complexes. These interactions are essential for the integration and modulation of immune responses [24,25].

Interestingly, we identified 454 TNL genes, which previous studies suggested did not exist in rice. Further experiments are needed to confirm whether these genes are indeed TNL genes. In summary, our study is the first to utilize high-quality rice genomes for a systematic identification of LRR and NLR genes in rice, providing new insights for functional genomics research in rice, offering new data for the exploration of disease resistance genes, and presenting new strategies for disease-resistant breeding in rice.

## 2. Materials and Methods

### 2.1. Collection and Organization of Rice Genome Information

This study encompassed a total of 300 plant genomes, with the data primarily sourced from three distinct research studies [1,2,3]. Detailed sample information can be found in Supplementary Table S1. For each gene, the corresponding genome sequence files (.fa format) and annotation files (.gff3 format) were downloaded. For each genome, the coding gene protein sequences and CDS sequences (both in .fa format) were extracted using gffread (Version 0.12.7) [26]. For each gene, if multiple transcripts were available, the longest transcript was selected for subsequent analyses. The 300 genomes underwent rigorous quality benchmarking with Benchmarking Universal Single-Copy Orthologs (BUSCO) to assess their assembly integrity. The BUSCO score evaluates genomic data integrity by analyzing complete, fragmented, and missing genes. High completeness and low missing rates reflect superior data quality, supporting applications in genomic quality control, gene prediction, comparative/phylogenomic analyses, and metagenomics [27].

### 2.2. Identification of LRR Genes

The leucine-rich repeat (LRR) gene is a part of the plant nucleotide-binding leucine-rich repeat (NLR) receptor and is responsible for recognizing pathogen effectors, thereby triggering the ETI immune response [25]. NLR identification was performed following the methodology outlined in Ngou et al. [28]. In summary, protein sequences shorter than 250 amino acids were removed, and the remaining protein sequences were subjected to domain searches using HMMER (Version 3.1b2). The threshold for filtering protein kinase domains was set at 1 × 10^−10^, while the threshold for LRR domains was set at 10 × 10^−3^. For protein kinase domains, the focus was primarily on Protein Families Database (PFAM) PF00069.26, whereas for LRR domains, the relevant PFAM entries included PF18805.2, PF18831.2, PF18837.2, PF00560.34, PF07723.14, PF07725.13, PF12799.8, PF13306.7, PF13516.7, PF13855.7, PF14580.7, PF01463.25, PF08263.13, and PF01462.19. Genes containing both protein kinase and LRR domains were classified as LRR receptor-like kinases (LRR-RLKs). Genes containing LRR domains but lacking protein kinase domains and possessing C3F domains were classified as Leucine-rich repeat receptor-like proteins (LRR-RLPs). Additionally, lysin motif receptor-like kinases (LysM-RLKs) and lysin motif receptor-like proteins (LysM-RLPs) were filtered based on protein sequence lengths greater than 150 amino acids. Protein sequences containing both LysM domains (PF01476.21) and transmembrane domains were classified as *LysM* genes. Further classification of *LysM* genes into LysM-RLKs and LysM-RLPs was conducted based on the presence of kinase domains. The software used for identifying *LysM* genes included TransMembrane prediction using hidden Markov models (TMHMM) (Version 2.0) [29].

### 2.3. Identification of NLR Genes

For the identification of NLR genes, we utilized NLR-Annotator (Version 2) [30], a software widely used for NLR gene identification in plants. All parameters were set to their default values, and further grouping analyses were conducted based on the results provided by the software.

### 2.4. Construction of the Phylogenetic Tree for 300 Rice Genomes

To obtain the phylogenetic relationships among the 300 rice genomes, we conducted a comparative analysis of all protein sequences using OrthoFinder (Version 2.5.4) [31], with all parameters set to their default values. The phylogenetic tree output by OrthoFinder served as the phylogenetic tree for the 300 rice genomes. The online tool iTOL (https://itol.embl.de/ (accessed on 15 May 2025), Version 6) [32] and the R package itol.toolkit (Version 1.1.7) [33] were used for visualizing the phylogenetic tree.

### 2.5. Data Analysis and Visualization

All data analyses and visualizations in this study were conducted using R (Version 4.2.3) [34]. Data visualization was performed using the R package ggplot2 (Version 3.5.1) [35].

## 3. Results

### 3.1. Collection and Analysis of Rice Genome Samples

**Figure 1:**
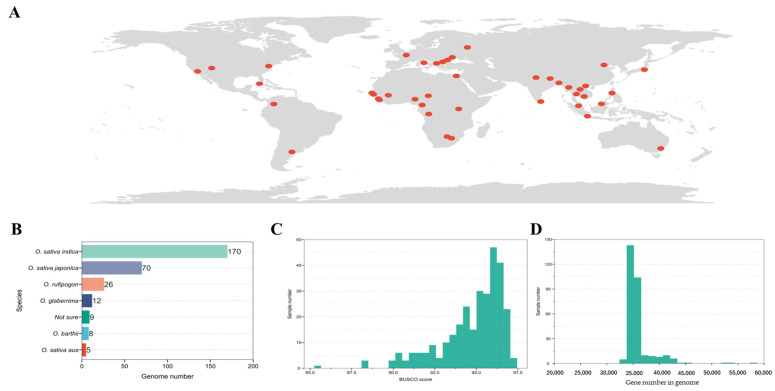
Distribution and description information of the 300 samples used in this study: (**A**) geographical distribution of samples; (**B**) subgroup identification and quantitative statistics; (**C**) BUSCO scores for 300 genomes; (**D**) distribution of gene counts in each genome. The red dots represent the geographical distribution where the sample sources are located.

To comprehensively explore the leucine-rich repeat (LRR) and nucleotide-binding leucine-rich repeat (NLR) in rice, we gathered genomic data from 300 rice samples sourced globally (Figure 1A), primarily from three studies [1,2,3]. Among these 300 rice genomes, 170 were from *Oryza sativa indica*, 70 from *O. sativa japonica*, 26 from *O. rufipogon*, 12 from *O. glaberrima*, 8 from *O. barthii*, 5 from *O. sativa aus*, and 9 could not be definitively assigned to a specific subgroup (referred to as “Not sure”) (Figure 1B). These genomes encompass the latest published T2T genome of rice. To ensure the accuracy of subsequent analyses, we conducted a quality assessment of the 300 genomes using Benchmarking Universal Single-Copy Orthologs (BUSCO) (Figure 1C). Only four genomes (NH268: 85.4, CN30: 88.1, CN14: 88.3, and CW02: 88.3) had BUSCO scores below 90, while the rest exhibited scores above 90, with an average of 94.78, a median of 95.40, and a maximum of 97.20. Regarding the number of genes in these genomes, the minimum was 33,308, the maximum was 57,359, the average was 35,919, and the median was 35,176 (Figure 1D). These results indicate that the 300 selected genomes represent the majority of the rice population and possess exceptionally high average quality, ensuring the precision of subsequent analyses.

### 3.2. Phylogenetic Tree and the Number of Each Gene Type

**Figure 2:**
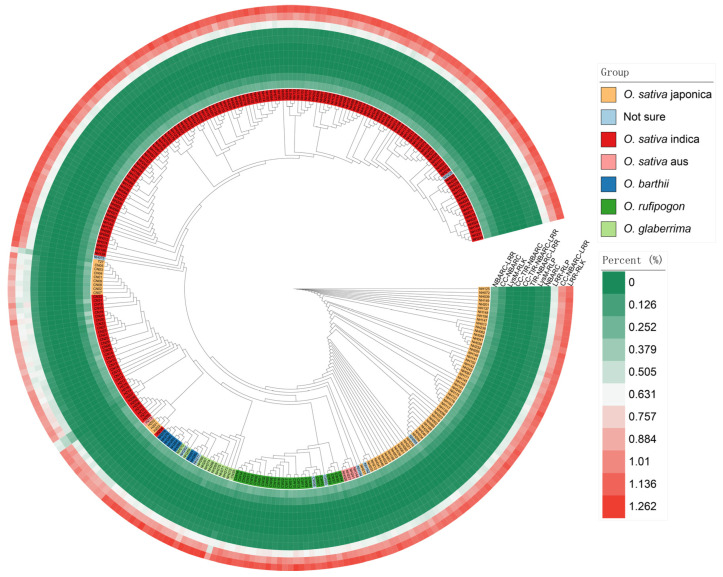
The phylogenetic tree of 300 rice accessions and the corresponding counts of NLR and LRR.

From Figure 2, it is evident that there are significant differences among the various subspecies. By comparing the proportion of each type of immune receptor gene, we can see that the number of LRR-RLK genes constitutes the highest percentage of the total gene count in the genome, followed by coiled-coil domain containing (CNL) genes and LRR-RLP genes. CNL-type NLRs contain a coil–coil (coiled coil, CC) domain, a nucleotide-binding domain (NB-ARC), and a leucine-rich repeat (LRR) [25]. Enhancing the expression of CNL-type NLR proteins in plants or optimizing their functionality via genetic engineering can substantially enhance crop disease resistance. For example, studies conducted on the model organism Arabidopsis thaliana have demonstrated that CNL-type R protein complexes, such as RRS1 and RPS4, recognize effectors from pathogenic bacteria, such as those causing bacterial blight, thereby activating plant immune responses and improving crop resistance to diseases [36].

### 3.3. Correlation Between the Sizes of Immune Receptor Families

**Figure 3:**
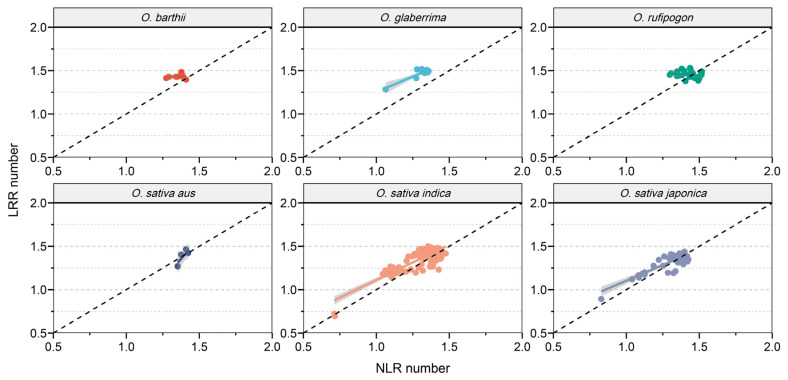
Correlation of the quantities of LRR and NLR within each subgroup.

Previous studies have revealed a synchronized expansion and contraction pattern of NLR and LRR in rice. In order to further investigate the relationship between NLR and LRR in different subgroups of rice, we compared their proportions relative to the total gene count (Figure 3). Interestingly, in *O. barthii* and *O. glaberrima*, both the quantity and proportion of LRR were significantly higher than those of NLR. In the other four subgroups, there was a significant positive correlation between the quantity and proportion of NLR and LRR. This result suggests that during the domestication process of *O. barthii* and *O. glaberrima*, LRR genes exhibited a more pronounced expansion effect compared to NLR genes, while in the other four subgroups, the contraction and expansion of LRR and NLR genes were similar.

### 3.4. Potential TNL Genes in Rice

**Figure 4:**
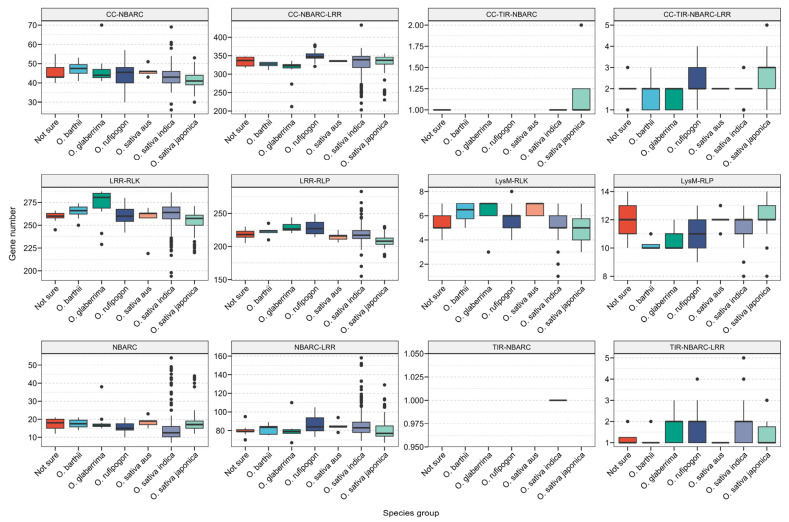
Subgroup information of NLR and LRR and their distribution across different rice subgroups.

Comparison revealed that, except for the coiled-coil Toll-interleukin-1 receptor–nucleotide-binding adaptor shared by APAF-1, R proteins, and CED-4 (CC-TIR-NBARC) and Toll-interleukin-1 receptor–nucleotide-binding adaptor shared by APAF-1, R proteins, and CED-4 (TIR-NBARC) subfamilies, the remaining LRR and NLR families were distributed across all rice subgroups (Figure 4). Interestingly, the TIR-NBARC subfamily was identified in *O. sativa indica*, distributed across five genomes (NH083, NH123, NH170, NH220, and NH246).

**Figure 5:**
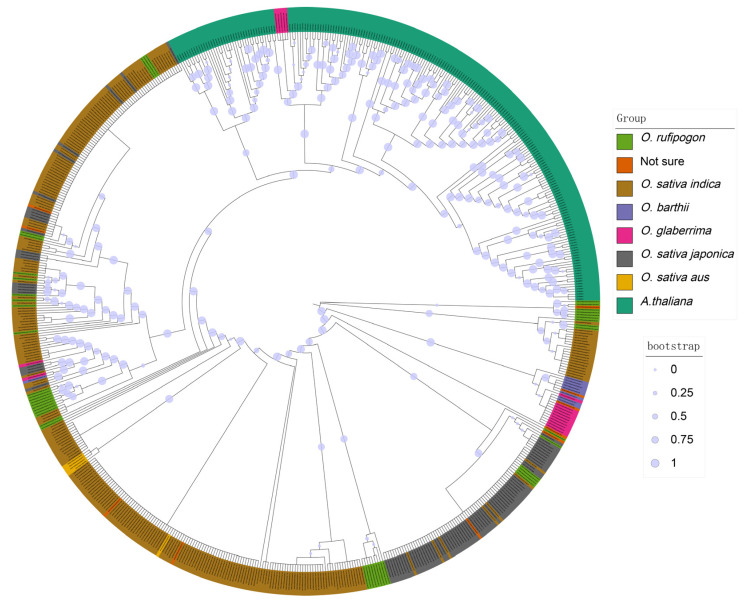
The phylogenetic tree of 454 rice TNL genes and 213 Arabidopsis TNL genes.

Typically, TNL genes are found only in dicotyledonous plants. However, in our study, NLR-Annotator identified 454 TNL genes distributed across all observed subgroups, with 275 in *O. sativa indica*, 91 in *O. sativa japonica*, 46 in *O. rufipogon*, 18 in *O. glaberrima*, 9 in *O. barthii*, 5 in *O. sativa aus*, and the remaining 10 distributed in the ‘not sure’ category. To confirm whether these TNL genes truly belong to the TNL gene category, we annotated NLR genes in the genome of Arabidopsis using NLR-Annotator, resulting in the identification of 213 TNL genes. Subsequently, a phylogenetic tree was constructed using the maximum likelihood method, combining these 213 confirmed TNL genes with the initial 454 potential TNL genes (Figure 5). From the tree, it is evident that Arabidopsis TNL genes cluster on a distinct evolutionary branch, while 5 out of the 454 rice TNL genes are interspersed with the Arabidopsis TNL genes cluster, all originating from the *O. glaberrima* subgroup and distributed across five accessions. To further confirm the nature of these 454 genes as TNL genes, we conducted NCBI CDD analysis on the protein sequences of the 667 genes. The results revealed that out of the 213 confirmed Arabidopsis TNL genes, 211 contained the PLN03210 domain. In contrast, among the 454 potential rice TNL genes, 293 contained the PLN03210 domain. The PLN03210 domain belongs to the PLN supergene family and typically encompasses one or more specific amino acid sequences that play a critical role in mediating protein–protein interactions [37,38]. This domain is likely involved in signal transduction processes in plants, particularly contributing significantly to responses against abiotic stress [38]. For instance, within the U-box gene family of Eucommia ulmoides, the PLN03210 domain co-occurs with other domains such as those from the Arm/Arm_2 superfamily and WD 40 superfamily, highlighting its importance in facilitating protein–protein interactions [37]. Interestingly, the five genes that were intermingled with Arabidopsis TNL genes did not contain the PLN03210 domain; instead, they harbored the GT1 domain along with the NB-ARC and LRR domains. Further comparison of the length of the PLN03210 domain between rice and Arabidopsis revealed an average length of 117 amino acids in rice and 986 amino acids in Arabidopsis for this domain. Additionally, these genes all contained the NB-ARC and LRR domains, with residues of the PLN03210 domain located at the C terminus. Collectively, these results indicate the presence of partial residues of the PLN03210 domain in rice, but complete TNL genes are not present. The residues of the PLN03210 domain, in conjunction with the NB-ARC and LRR domains, likely possess novel functionalities.

## 4. Discussion

The identification of a substantial number of LRR and NLR genes in our study underscores the complexity and robustness of the immune response in rice. These immune receptors are pivotal in recognizing pathogenic threats and initiating defense responses, which are critical for maintaining crop health and yield [7,8,9]. The similarity in gene counts across different rice subgroups suggests a conserved evolutionary strategy for combating biotic stresses [39]. This conservation may enable breeders to select for these immune receptor genes in various rice cultivars, enhancing the overall resilience of rice against a wide range of pathogens. Future research should focus on characterizing the specific functions of these genes and their interactions within the immune network to develop targeted breeding strategies.

The unexpected identification of 454 TNL genes in our analysis is particularly noteworthy (Figure 5), as previous studies had suggested their absence in rice [21]. This finding opens new avenues for research into the immune mechanisms of rice and challenges existing paradigms regarding its genomic landscape. TNL genes are known for their role in recognizing specific pathogen effectors and activating robust immune responses. Therefore, validating the functionality of these TNL genes could provide significant insights into rice’s defense strategies. The TIR-NB-LRR domain complex forms a platform for the recognition and interaction of effector proteins, leading to the activation of the immune response. The LRR domain specifically recognizes the effector proteins, while the TIR and NB domains coordinate the signal transduction process, ensuring a rapid and efficient immune response [24,40,41]. Moreover, understanding the evolutionary origins and adaptations of these genes could enhance our knowledge of plant immunity and inform comparative studies with other crops, potentially leading to broader applications in agricultural biotechnology.

The comprehensive genomic data generated from this study lay a solid foundation for future rice breeding programs aimed at enhancing disease resistance. By integrating genomic information with phenotypic data, breeders can employ marker-assisted selection to identify and propagate rice varieties with superior immune receptor profiles. Additionally, the insights gained from this research can guide the development of transgenic or gene-editing approaches to introduce or enhance specific immune receptor genes in rice. As global food security remains a pressing challenge, leveraging advanced genomic technologies to improve disease resistance in rice will be crucial in ensuring sustainable agricultural practices and securing food supplies for future generations.

## Data Availability

The data presented in this study are available in the article.

## Supplementary Materials

The following supporting information can be downloaded at: https://www.mdpi.com/article/10.3390/genes16050597/s1, Table S1. Genomic information of 300 rice samples.
